# Energy-Efficient Motion Simulation of a Bioinspired Variable Stiffness Joint Emulating Elbow Function for Periodic Tasks

**DOI:** 10.3390/biomimetics11070458

**Published:** 2026-07-01

**Authors:** Yapeng Xu, Kaishun Hao, Caidong Wang, Li Xiao, Wenming Wang

**Affiliations:** 1 College of Mechanical and Electrical Engineering, Zhengzhou University of Light Industry, Zhengzhou 450002, China; yapengxu@zzuli.edu.cn (Y.X.); 332404030342@zzuli.edu.cn (K.H.); 2 Engineering Training Centre, Zhengzhou University of Light Industry, Zhengzhou 450002, China; xiaoli2010@zzuli.edu.cn; 3 College of Mechanical and Transportation Engineering, China University of Petroleum-Beijing, Beijing 102249, China; wangwenming@cup.edu.cn

**Keywords:** elastic cantilever, variable stiffness joint, Archimedean spiral groove, resonance effect, energy consumption

## Abstract

Inspired by the energy-efficient resonance strategy of the human elbow joint during periodic arm swing, this paper investigates the energy-saving motion and performance of a robotic variable stiffness joint. A modular stiffness adjustment mechanism with continuously adjustable stiffness based on Archimedean spiral grooves is proposed. A co-simulation model using MATLAB (R2022b)/ADAMS (2020) is established, and dynamic equations are derived to reveal the correlation between resonance/anti-resonance frequencies and joint rotational stiffness. Mimicking the biological principle of stiffness-frequency matching, an energy-saving controller leveraging the resonance effect is designed, which includes a motor energy consumption model to quantify losses and an optimization strategy to match the joint rotational stiffness with the load anti-resonance frequency. Simulation results demonstrate that in variable stiffness mode, aligning the system anti-resonance frequency with the task trajectory frequency significantly reduces joint energy consumption, validating the bioinspired approach. In contrast, the high-stiffness (rigid) mode leads to a surge in system energy consumption.

## 1. Introduction

Arm swing during walking or running plays an important role in balance and energy expenditure in humans [1,2]. For robotic systems performing similar periodic motions, traditional rigid actuators struggle to optimize energy consumption, especially in periodic motion [3]. In nature, however, biological joints, such as the human elbow, effortlessly achieve energy-efficient periodic motion by modulating joint rotational stiffness to resonate with movement rhythms [4]. Variable stiffness actuators (VSAs) represent an engineering embodiment of this biological principle, enabling dynamic joint rotational stiffness adjustment to mimic such natural efficiency. Guided by this bio-inspired perspective, existing research on the energy-saving performance of VSAs can be broadly categorized into mechanism configuration design, application-driven development, novel structural innovations, and control-oriented approaches [5,6,7].

Several design philosophies have been explored to reduce energy consumption. One approach focuses on the stiffness adjustment mechanism itself by relocating heavy components (e.g., the adjustment motor) to the base [8] or eliminating high-friction elements such as gearboxes and ball screws via linkage mechanisms [9], significantly reducing inertia and parasitic energy draw. Another strategy targets elastic element design: adaptive torsion springs that compensate motion-induced torque [10], variable leaf spring length [11,12], and cam mechanisms that avoid spring preload adjustment losses [13] have demonstrated superior efficiency. Parallel variable stiffness configurations, achieved via multi-spring combinations or fulcrum adjustments, reduce peak power demand [14]. Further refinements in friction loss reduction and elastic element layout lower operational energy consumption [15]. Mechanical compensation and cam profiles that maintain static stability at the balance position enable low-power stiffness switching [16]. Beyond individual components, a design for tuning stiffness parameters, combined with energy-efficiency-oriented optimization, significantly improves actuator energy utilization [17].

Research on legged robots, rehabilitation assistance, and prosthetic exoskeletons further highlights the engineering value of VSA energy-saving performance. Biomechanical requirements of human lower limb joints during daily activities and sports tasks provide scene adaptation baselines for energy consumption design [18]. Variable stiffness ankle exoskeletons [19], intermittent VSAs (iVSA) [20], and dynamically driven prosthetic knees [21] have all demonstrated reduced motor torque and power demand. Leaf spring-based ankle foot prostheses offer low mass, power, and cost [22]. Biologically inspired stiffness optimization strategies reduce human metabolic cost when wearing exoskeletons [23], while adaptive stiffness control strategies improve joint energy efficiency [24]. Other designs include soft hard hybrid pneumatic rotary VSAs for extreme environments [25], tendon-driven VSAs based on adjustable cantilever mechanisms [26], and passive flexible joints with series parallel elastic elements [27].

Recent years have seen a variety of new variable stiffness joints broadening stiffness adjustment range, improving structural integration, and adapting to complex environments. These include an electromagnetically driven compact VSA [28], a trapezoidal-screw-based actuator with high response speed and wide stiffness range [29], a modular VSA based on equivalent lever and planetary gear transmission [30], a rocker linkage planetary gear actuator with linear guides and cam followers to reduce friction loss [31], and a compact VSA using a variable-radius pulley spring mechanism [32].

The nonlinearity, hysteresis, disturbance, and flexible vibration problems inherent to variable stiffness systems have also received widespread attention. Control strategies include reinforcement learning for stiffness control in 4D-printed VSAs [33], adaptive sliding mode control (SMC) based on barrier functions [34], dynamic surface backstepping position control combined with radial basis function neural networks and disturbance observers (DSBC-RBFNN-DOB) [35], Maxwell-slip-based correction models to compensate hysteresis-induced torque errors [36], unknown-input-observer (UIO)-based torque control systems to address output vibration [37], and fault-tolerant control strategies for output trajectory tracking in elastic actuators [38].

**Figure 1:**
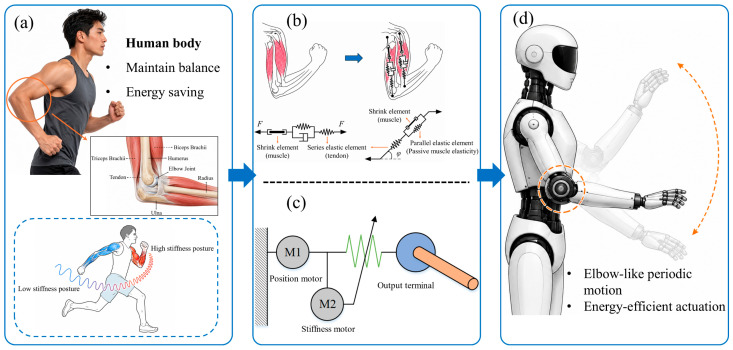
Bioinspired rationale of the proposed variable stiffness joint. (**a**) The muscle state during human elbow joint movement.; (**b**) Abstract muscle drive model of elbow joint.; (**c**) Principle of variable stiffness actuation; (**d**) The application prospects of humanoid robot elbow joint.

Biologically, the human elbow joint provides a compelling paradigm for energy-efficient periodic motion. As shown in Figure 1a, during activities such as walking or running, the antagonistic muscle pairs (biceps and triceps) co-activate to modulate joint rotational stiffness, thereby tuning the natural frequency of the forearm to resonate with the locomotion rhythm [39]. This resonance effect minimizes active energy input by allowing gravitational forces and tendon elasticity to passively drive most of the swing cycle, significantly reducing metabolic energy consumption [7]. As shown in Figure 1b,c, it abstracts this biological mechanism into a musculoskeletal model with antagonistic muscle-tendon units, which is then mapped to an engineering variable-stiffness actuator model where stiffness can be modulated by adjusting antagonistic actions or mechanical design parameters. This abstraction bridges the biological principle of matching system stiffness to task frequency to exploit resonance for energy savings to a realizable robotic joint design, which also has practical significance for energy-saving control of humanoid robots, as shown in Figure 1d.

Inspired by this resonance-driven energy efficiency principle of the human elbow, this paper investigates the energy-saving motion characteristics of a robotic variable stiffness joint driven by a leaf-spring-based stiffness regulation mechanism. A modular joint structure using Archimedean spiral grooves is designed to achieve continuous stiffness variation by changing the effective leaf spring length. Based on the proposed mechanism, the dynamic behavior of the joint is modeled, and the influence of stiffness variation on the resonance and antiresonance characteristics of the system is analyzed. To reduce energy consumption during periodic motion, a stiffness optimization strategy is developed by aligning the system antiresonance frequency with the dominant frequency of the desired trajectory. The effectiveness of the proposed method is evaluated by ADAMS/MATLAB co-simulation, with particular attention paid to the comparison of energy consumption under rigid, constant stiffness, and variable stiffness modes.

As shown in Table 1, a variety of spring-based VSA systems have been developed using distinct stiffness adjustment mechanisms. Stiffness ranges vary considerably across designs, from narrow-band systems below 30 Nm/rad to wide-range designs exceeding 1000 Nm/rad, with structural complexity generally scaling with achievable bandwidth. Notably, most existing studies focus primarily on mechanical design and stiffness characterization, while only a limited number explicitly investigate energy-saving control strategies for periodic motions [9,10,11,13]. The dominant approaches rely on passive energy storage and release or low-power stiffness regulation [8,12,28], with few exploring explicit matching between joint stiffness and system resonance characteristics [13]. In comparison, the proposed VSJ offers a stiffness range from 11 Nm/rad to near infinity, comparable to or broader than most existing designs, while maintaining moderate structural complexity. More importantly, unlike most previous works that treat variable stiffness primarily as a mechanical functionality, the present study establishes a direct analytical relationship between joint stiffness and anti-resonance frequency, and develops a bioinspired stiffness-frequency matching strategy that leverages the resonance effect to minimize actuator power during periodic motions. This principle, inspired by human elbow resonance during rhythmic swinging, has received limited attention in the context of Archimedean spiral-based VSJ designs. Thus, the primary novelty lies in the theoretical formulation of a stiffness-tuning framework that extends the role of VSJs from passive adaptability to active energy optimization in periodic tasks.

The organization of this paper is as follows. Section 2 describes the prototype structure of the variable-stiffness joint, explains its stiffness adjustment principle, and derives the corresponding dynamic model. Section 3 develops the energy-saving motion control framework, including trajectory tracking control, motor energy consumption modeling, and stiffness regulation based on anti-resonance frequency matching. Section 4 presents the simulation studies for periodic motion and analyzes the energy-saving performance of the joint under different stiffness modes. Section 5 discusses the applicable scenarios and limitations of the proposed method. Section 6 concludes the paper by summarizing the main findings and the potential value of the proposed variable-stiffness joint for robotic energy optimization.

## 2. Robot Variable Stiffness Joint Modeling

In periodic robotic operations, energy consumption is a key actuator performance metric. Traditional rigid joints lack the flexibility to adapt to dynamic trajectories, often requiring additional torque to compensate for tracking errors, leading to high energy consumption. Series elastic actuators (SEAs) offer inherent flexibility, but their fixed stiffness cannot match trajectory frequency dynamics, resulting in considerable energy waste in non-resonant bands. In contrast, variable stiffness joints (VSJs) enable effective stiffness adjustment, providing a structural basis for energy-saving control under dynamic trajectories. This paper presents a simulation study of the motion and energy consumption behavior of a VSJ to support energy-efficient control strategy design. The working principle of the proposed joint is first described.

### 2.1. Basic Principle of Variable Stiffness Joint

**Figure 2:**
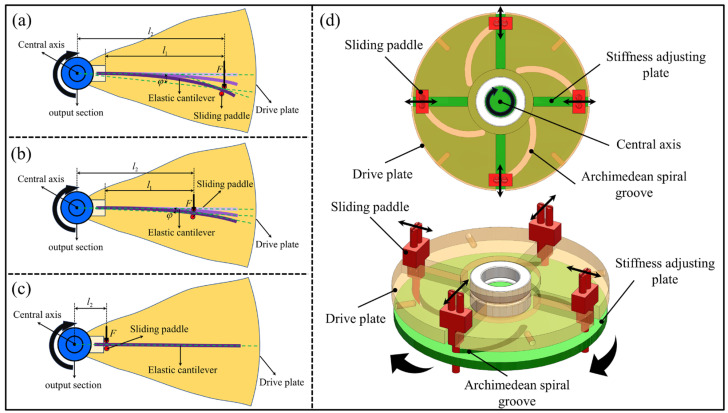
Principle of stiffness adjustment based on changing the effective length of elastic cantilever (leaf spring). (**a**) Low stiffness mode. (**b**) Medium stiffness mode. (**c**) High stiffness mode. (**d**) Sliding paddle regulation by Archimedes spiral groove.

The basic variable stiffness principle of the proposed VSJ is based on changing the effective length of the elastic cantilever (leaf spring). As shown in Figure 2a, one leaf spring is placed in the VSJ as an example, and one end is fixed to the output section, forming an elastic cantilever beam state. The sliding paddle is installed on the drive plate below and in contact with the elastic cantilever. The drive plate, output section, and elastic cantilever can all rotate around the central axis. When the drive plate rotates clockwise with the sliding paddle, it will generate a load force *F* on the elastic cantilever, causing it to bend and deform, thereby driving the output section to rotate, forming the so-called series elastic actuation. If the relative position of the sliding paddle on the elastic cantilever is changed, the effective suspension length *l*_1_ of the elastic cantilever will be altered. As shown in Figure 2a–c, when the sliding paddle moves from the free end of the elastic cantilever to the fixed end, the effective suspension length of the elastic cantilever will be shortened, and the corresponding transmission stiffness between the drive plate and the output section will also increase from the minimum to an approximate rigid mode, ultimately achieving joint rotational stiffness adjustment. The movement of the sliding paddle along the elastic cantilever is essentially a radial movement within the entire VSJ. As shown in Figure 2d, in order to improve the response speed and avoid the complexity of the stiffness adjustment mechanism, the stiffness adjusting plate and the drive plate are used in combination with differential motion, and the Archimedes spiral groove on the stiffness adjusting plate drives the sliding paddle to move radially in a straight line on the drive plate.

**Figure 3:**
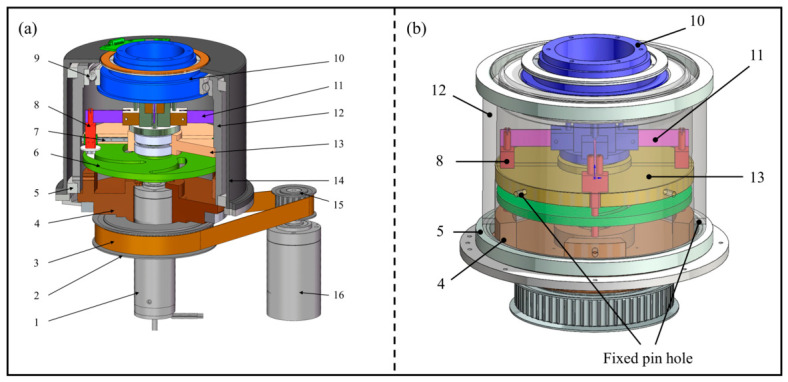
3D model of the variable stiffness joint. 1—stiffness motor, 2—large pulley, 3—drive belt, 4—drive end plate, 5—bearing, 6—stiffness adjustment plate, 7—slider, 8—sliding paddle, 9—bearing, 10—output end plate, 11—leaf spring (elastic cantilever), 12—main drive shaft frame, 13—drive plate, 14—housing, 15—small pulley, 16—position motor. (**a**) Overall partial sectional view; (**b**) The overall assembly relationship of the input module.

Based on the previously designed variable stiffness joint [40], the parallel leaf spring layout is adopted to form the main mechanism for joint rotational stiffness adjustment. As shown in Figure 3, the position motor 16 drives the small pulley 15 and transmits the power to the large pulley 2 and the drive end plate 4 through the drive belt 3. The drive end plate 4 and drive plate 13 are coaxially fixed on the main drive shaft frame 12 by the pin holes distributed along the sides. The stiffness motor 1 is also coaxially installed at the center of the drive end plate 4, with its output shaft used to drive the stiffness adjustment plate 6. The stiffness adjustment plate 6 is coaxially installed and supported on the other side of the drive end plate 4 through bearings, and can rotate relative to the drive plate 13 under the drive of the stiffness motor 1. But the stiffness adjustment plate 6 does not have a direct constraint relationship with the drive plate 13. Leaf spring (elastic cantilever) 11 is uniformly installed on the component where the output end plate 10 is located and rotates together with it. The output end plate 10 is supported by bearings and installed on the inner side of the main drive shaft frame 12. Since the drive end plate 4 is fixedly connected with the main drive shaft frame 12, the sliding paddle 8 then squeezes the leaf spring on the output shaft and finally transmits the driving force to the output end plate 10. The three-dimensional model of the robot VSJ is mainly composed of three parts: spindle drive, stiffness adjustment and shell, which are connected in series.

The elastic torque τ acting on the joint can be calculated as follows:

(1) $$\tau =NFl_{2}\cos\varphi =\frac{3EIKl_{2}\tan\varphi }{l_{1}^{3}}Nl_{2}\cos\varphi =\frac{NEbh^{3}l_{2}^{2}\sin\varphi }{4l_{1}^{3}}$$

Therefore, the total joint rotational stiffness can be expressed as:

(2) $$K_{j}=\frac{d\tau }{d\varphi }=\frac{NEbh^{3}l_{2}^{2}\cos\varphi }{4l_{1}^{3}}$$

where N is the leaf spring number participating in the stiffness adjustment, E is the Young modulus of the leaf spring, b and h represent the width and thickness of the leaf spring, $l_{2}$ is the distance from the action point of leaf spring to the output shaft axis, φ is the joint elastic deflection angle, and $l_{1}$ is the effective cantilever length leaf spring.

**Figure 4:**
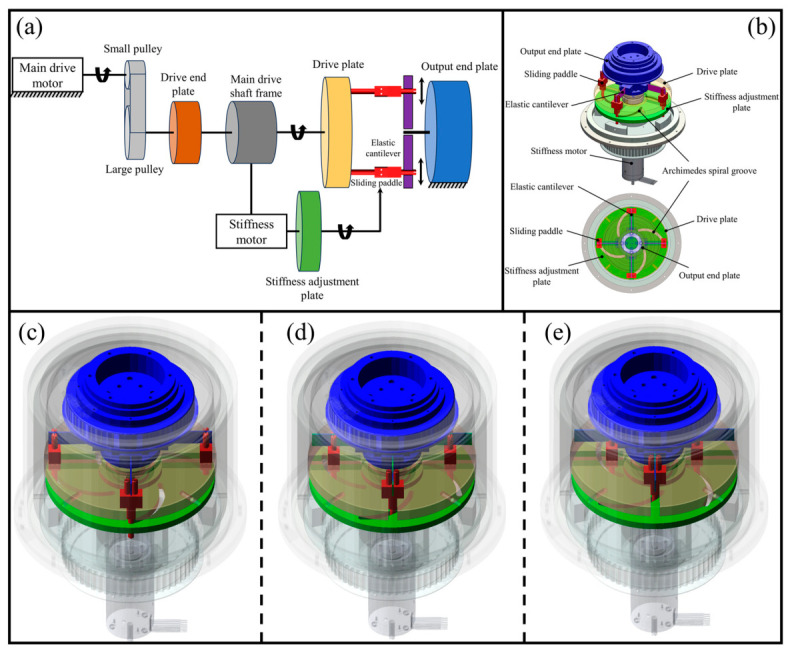
Joint kinematics diagram and joints under different stiffness states. (**a**) Joint kinematics diagram. (**b**) Internal view of joint. (**c**) Joint in low stiffness state. (**d**) Joint in medium stiffness state. (**e**) Joint in high stiffness state.

Based on the joint rotational stiffness model, Figure 4 shows the VSJ kinematic configuration under different stiffness settings. As shown in Figure 4a, the large pulley, drive end plate, main drive shaft frame, drive plate, sliding paddle, stiffness motor, and stiffness adjustment plate are coaxially mounted and rotate synchronously, forming the total input shaft. Power from the main drive motor is transmitted through this input shaft via a synchronous belt, then through the elastic cantilever (leaf springs), and finally to the output end plate, creating a series elastic actuation path. In the local stiffness adjustment mechanism, the stiffness adjustment plate can rotate differentially relative to the drive plate to radially reposition the sliding paddles. As shown in Figure 4b, the Archimedes spiral grooves convert this relative rotation into radial displacement of the paddles, which serve as movable contact points for the leaf springs. Their radial positions directly determine the effective cantilever length of each spring. Figure 4c–e depict the internal joint states under low-, medium-, and high-stiffness modes, respectively.

Based on the above stiffness adjustment principle and transmission scheme, the core parameters of the VSJ designed are shown in Table 2, where the weight of the VSJ is calculated by configuring the corresponding part materials in SolidWorks (2023) 3D software. The stiffness adjustment range is calculated theoretically using Equation (2) and the effective adjustment length *l*_1_ of the leaf spring.

### 2.2. Dynamic Modeling of Variable Stiffness Joint

**Figure 5:**
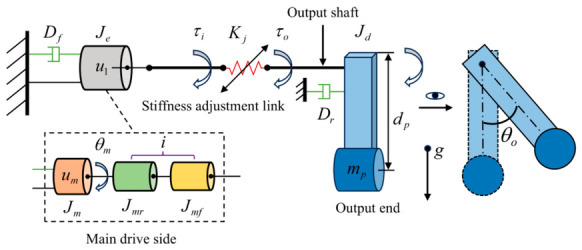
Simplified model of the VSJ dynamics.

The variable stiffness joint is composed of the main drive structure, elastic cantilever and output shaft. The stiffness adjustment mechanism is relatively independent, and the two transmission chains can operate independently. The simplified model of VSJ dynamics is shown in Figure 5, which includes the main drive link and the stiffness adjustment link. The joint torque $\tau_{o}$ can be defined as:

(3) $$\tau_{o}=K_{j}\varphi =K_{j}\left(\theta_{i}-\theta_{o}\right)$$

where $\theta_{i}$ and $\theta_{o}$ represent the input and output side angles, respectively. The relationship between the position motor angle $\theta_{m}$ and the output side angle $\theta_{o}$ is defined as:

(4) $$\theta_{m}=i\left(\frac{\tau_{o}}{K_{j}}+\theta_{o}\right)$$

where i is the reducer gear ratio.

The main driving dynamic model of the VSJ can be defined as:

(5) $$\left\{\begin{array}{c} J_{d}{\ddot{\theta }}_{o}+D_{r}{\dot{\theta }}_{o}+G\left(\theta_{o}\right)=\tau_{o}\left(\varphi ,l_{1}\right)\\ J_{e}{\ddot{\theta }}_{i}+D_{f}{\dot{\theta }}_{i}+\tau_{o}\left(\varphi ,l_{1}\right)=u_{1}(t) \end{array}\right.$$

where $\theta_{i}=\theta_{m}/i$, $G\left(\theta_{o}\right)=m_{p}gd_{p}\sin\left(\theta_{o}\right)$ is the output side gravity, and $d_{p}$ is the force arm length of the load mass $m_{p}$. $J_{e}=iJ_{m}+iJ_{mr}+J_{mf}$, $J_{m}$ is the position motor inertia, $J_{mr}$ is the reducer inertia, $J_{mf}$ is the inertia of the input shaft frame, $J_{d}$ is the total kinematic inertia of the output end, $D_{f}$ and $D_{r}$ represent the damping coefficients of the input and output ends, respectively. $u_{1}(t)$ is the control torque of the main drive mechanism, which is calculated online by the controller.

Considering that there must be a phase difference between the motor end of the main drive and the output end of the joint, the transmission efficiency of the reduction mechanism can be defined as:

(6) $$C=\left\{\begin{array}{r} 1/\eta_{tr},\;\;\;\left(u_{m}-\left(J_{m}+J_{mr}\right){\ddot{\theta }}_{m}\right){\dot{\theta }}_{m}\geq 0,\\ \left(\mathrm{Motor}\;\mathrm{drive}\;\mathrm{load}\right)\\ \eta_{tr},\;\;\;\;\;\;\;\left(u_{m}-\left(J_{m}+J_{mr}\right){\ddot{\theta }}_{m}\right){\dot{\theta }}_{m}<0,\\ \left(\mathrm{Load}\;\mathrm{drive}\;\mathrm{motor}\right) \end{array}\right.$$

where $u_{m}$ is the position motor torque and $\eta_{tr}$ is the transmission efficiency of the reduction mechanism.

According to the above analysis, the detailed dynamic model of the VSJ can be expressed as:

(7) $$\left\{\begin{array}{l} \tau_{o}-K_{j}\left(\frac{\theta_{m}}{i}-\theta_{o}\right)=0\\ \left(iJ_{m}+iJ_{mr}+J_{mf}\right)\frac{{\ddot{\theta }}_{m}}{i}+\\ \frac{C}{i}\left[K_{j}\left(\frac{\theta_{m}}{i}-\theta_{o}\right)+D_{f}\frac{{\dot{\theta }}_{m}}{i}\right]=u_{m} \end{array}\right.$$

The definition of the reducer mechanism efficiency is the same as Equation (6). Here is the case of the motor-driven load:

(8) $$\left(u_{m}-\left(J_{m}+J_{mr}\right){\ddot{\theta }}_{m}\right){\dot{\theta }}_{m}\geq 0$$

This indicates a positively driven chain with non-negative power. To facilitate discussion, by ignoring all damping and linearizing the gravity term, the two equations in (7) can be combined as follows:

(9) $$(iJ_{m}+iJ_{mr}+J_{mf})\frac{{\ddot{\theta }}_{m}}{i}+\frac{C}{i}\left(J_{d}{\ddot{\theta }}_{o}+m_{p}gd_{p}\theta_{o}\right)=u_{m}$$

Therefore, the second derivative of Equation (4) can be obtained, and ${\ddot{\theta }}_{m}$ is brought into Equation (9). The result is transformed into frequency-domain analysis, and the transfer function $H_{u_{m}}^{\theta_{o}}\left(\omega \right)$ from motor torque to the output angle of the VSJ is defined as:

(10) $$H_{u_{m}}^{\theta_{o}}\left(\omega \right)=\frac{\theta_{o}}{u_{m}}=\frac{K_{j}}{c_{4}\omega^{4}+c_{2}\omega^{2}+c_{0}}$$

The coefficients are defined as:

(11) $$\left\{\begin{array}{cc} c_{4} & =J_{d}(iJ_{m}+iJ_{mr}+J_{mf})=J_{e}J_{d}\\ c_{2} & =(iJ_{m}+iJ_{mr}+J_{mf})(m_{p}gd_{p}+K_{j})+CK_{j}J_{d}/i\\ & =J_{e}(m_{p}gd_{p}+K_{j})+CK_{j}J_{d}/i\\ c_{0} & =CK_{j}m_{p}gd_{p}/i \end{array}\right.$$

This is a fourth-order transfer function, and its two poles correspond to the two resonant frequencies of the system:

(12) $$\omega_{r1,2}=\pm {\left(\frac{-c_{2}\pm \sqrt{c_{2}^{2}-4c_{4}c_{0}}}{2c_{4}}\right)}^{1/2}$$

Similarly, according to Equations (7) and (9), the transfer function between motor angle and motor torque can be obtained as:

(13) $$H_{u_{m}}^{\theta_{m}}\left(\omega \right)=\frac{\theta_{m}}{u_{m}}=\frac{-iJ_{d}\omega^{2}+i(m_{p}gd_{p}+K_{j})}{c_{4}\omega^{4}+c_{2}\omega^{2}+c_{0}}$$

The coefficients $c_{4}$, $c_{2}$ and $c_{0}$ are consistent with those in Equation (11). The zero point of this transfer function is:

(14) $$\omega_{a}=\sqrt{\frac{m_{p}gd_{p}+K_{j}}{J_{d}}}$$

where $\omega_{a}$ corresponds to the anti-resonant frequency of the joint system, which can be called the natural frequency of the output side. At this frequency, fixing the main drive motor results in a peak oscillation at the output end. Since mechanical power is proportional to speed, the anti-resonant frequency $\omega_{a}$ could be exploited as a beneficial working point for the joint, similar to resonance. According to Equation (14), if the joint rotational stiffness $K_{j}$ is actively adjusted, the natural frequency of the system will change.

**Figure 6:**
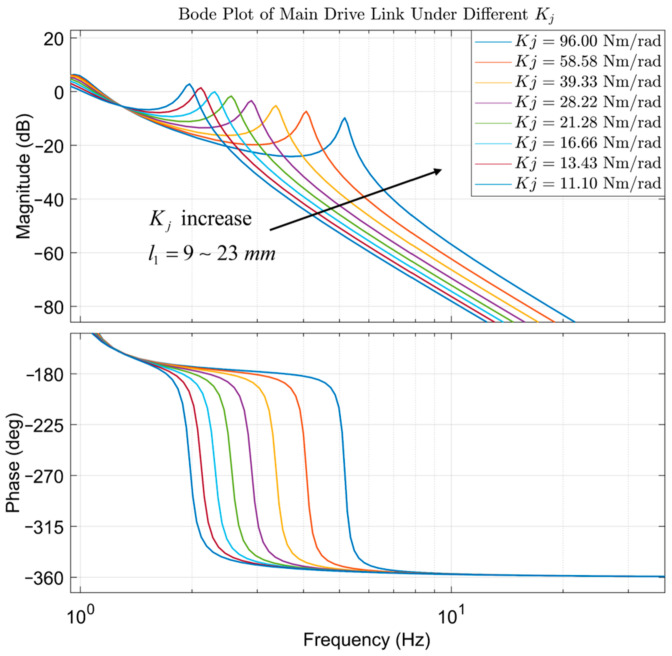
Amplitude-frequency characteristics of the VSJ main driving link under varying stiffness.

According to Figure 6, with the increase in stiffness $K_{j}$, the amplitude frequency curve form of the system moves to the high-frequency region. The natural frequency increases, and the bandwidth expands, reflecting the direct shaping effect of stiffness adjustment on the dynamic characteristics of the system. The rapid attenuation of high-frequency amplitude indicates that the joint has good vibration suppression and energy absorption ability at high frequencies. The results verify that VSJ can achieve continuous adjustment of the natural frequency of the output side through active stiffness adjustment, so as to achieve dynamic adjustment between response speed and compliance.

## 3. Controller Design for Energy Saving Motion

The structural design and dynamic modeling of the variable stiffness joint (VSJ) reveal that by adjusting the effective length of the elastic cantilever, the joint output stiffness can be continuously varied, enabling dynamic matching between the system’s natural frequency and the load motion frequency to achieve resonance-based energy savings. A position control strategy is first designed to suppress flexible vibrations, ensuring trajectory-tracking accuracy. Subsequently, a motor energy consumption model is constructed to quantify energy losses under various operating conditions. Finally, a stiffness optimization control method is developed to enable real-time matching of the joint rotational stiffness with the system’s anti-resonance frequency, forming an integrated control strategy that balances both accuracy and energy efficiency.

### 3.1. Position Control for VSJ

**Figure 7:**
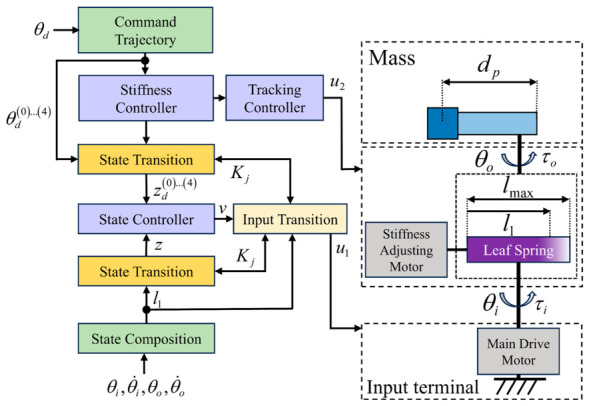
VSJ basic transmission and control architecture.

As shown in Figure 7, the basic transmission and control architecture of the VSJ is established. The output torque of the stiffness adjustment motor drives the adjusting disc to rotate, thereby changing the stress position and effective spring length to achieve continuous joint rotational stiffness adjustment. The input torque of the position motor can be calculated, allowing the joint rotational stiffness to be adjusted as required. Furthermore, the joint features reconfigurability of the stiffness adjustment range. By manually rotating the screw corresponding to each elastic cantilever, the number of elastic cantilevers participating in stiffness adjustment can be varied, enabling reconstruction of the overall stiffness range. This enhances the system’s adaptability to different loads and motion conditions.

Referring to Figure 5, the moment of inertia of the pendulum at the output end is $J_{d}$, the reduced moment of inertia of the position motor, and the reduction mechanism and input shaft frame at the drive end is $J_{e}=i\left(J_{m}+J_{mr}\right)+J_{mf}$. Referring to Figure 7, Equation (5) can be rewritten by a matrix expression in the nonlinear state space as:

(15) $$x={\left[{\begin{array}{cccc} x_{1} & x_{2} & x_{3} & x \end{array}}_{4}\right]}^{T}={\left[\begin{array}{cccc} \theta_{o} & {\dot{\theta }}_{o} & \theta_{i} & {\dot{\theta }}_{i} \end{array}\right]}^{T}$$

Therefore, the joint system can be represented by:

(16) $$\dot{x}=Ax+Bu_{1}+{\left[0\;-\frac{m_{p}gd_{p}}{J_{d}}\sin\left(x_{1}\right)\;0\;0\right]}^{T}$$

where $A=\left[\begin{array}{cccc} 0 & 1 & 0 & 0\\ -\frac{K_{j}}{J_{d}} & 0 & \frac{K_{j}}{J_{d}} & 0\\ 0 & 0 & 0 & 1\\ \frac{K_{j}}{J_{e}} & 0 & -\frac{K_{j}}{J_{e}} & 0 \end{array}\right]$, $B={\left[\begin{array}{cccc} 0 & 0 & 0 & \frac{1}{J_{e}} \end{array}\right]}^{T}$, $u_{1}\left(x,z\right)=K_{j}^{-1}(J_{e}J_{d})\left[v(z)-a(x)\right]$ is equivalent to input torque $\tau_{i}$, and a(x) is a nonlinear feedback term which is defined as:

(17) $$a(x)=\frac{m_{p}gd_{p}}{J_{d}}\sin(x_{1})\left[x_{2}^{2}+\frac{m_{p}gd_{p}}{J_{d}}\cos(x_{1})+\frac{K_{j}}{J_{d}}\right]+\frac{K_{j}}{J_{d}}(x_{1}-x_{3})\left[\frac{K_{j}}{J_{d}}+\frac{K_{j}}{J_{e}}+\frac{m_{p}gd_{p}}{J_{d}}\cos(x_{1})\right]$$

The new input v(z) can be written as:

(18) $$z=L{\left[\begin{array}{cccc} x_{1} & x_{2} & x_{3} & x_{4} \end{array}\right]}^{T}+{\left[\begin{array}{cccc} 0 & 0 & \frac{m_{p}gd_{p}}{J_{d}}\sin\left(x_{1}\right) & \frac{m_{p}gd_{p}}{J_{d}}x_{2}\cos\left(x_{1}\right) \end{array}\right]}^{T}$$

where $L=\left[\begin{array}{cccc} 1 & 0 & 0 & 0\\ 0 & 1 & 0 & 0\\ -\frac{K_{j}}{J_{d}} & 0 & \frac{K_{j}}{J_{d}} & 0\\ 0 & -\frac{K_{j}}{J_{d}} & 0 & \frac{K_{j}}{J_{d}} \end{array}\right]$.

For this system, the tracking control law is designed as:

(19) $$v\left(z\right)=z_{d}^{\left(4\right)}+k_{1}\left(z_{d,1}-z_{1}\right)+k_{2}\left(z_{d,2}-z_{2}\right)+k_{3}\left(z_{d,3}-z_{3}\right)+k_{4}\left(z_{d,4}-z_{4}\right)$$

where $z_{d}^{\left(4\right)}={\ddddot{\theta }}_{d}$ represents the fourth derivative of the command trajectory, $z_{1}$ to $z_{4}$ correspond to the output position $\theta_{o}$, velocity ${\dot{\theta }}_{o}$, acceleration ${\ddot{\theta }}_{o}$ and jerk ${\dddot{\theta }}_{o}$, respectively. $z_{d,1}$ to $z_{d,4}$ correspond to the command input position, velocity, acceleration and jerk. $k_{1}$ to $k_{4}$ correspond to its control gain, respectively.

The key dynamic parameters used in the co-simulation are listed in Table 3. These values are derived from multiple sources according to their physical nature. The inertial parameters, including input inertia *J_e_*, the output inertia *J_d_*, load inertia (calculated from *m_p_* and *d_p_*), and the gear ratio *i*, are determined based on the mechanical configuration defined in the 3D CAD model (SolidWorks) and the specified load conditions. The limits of elastic cantilever length are directly obtained from the geometric constraints of the Archimedean spiral groove design. The damping coefficients of *D_r_* and *D_f_*, as well as the reduction mechanism efficiency, are adopted from representative studies on VSJ/VSA [41,42] and further calibrated through least-squares fitting against the dynamic response characteristics of the simulation model. The controller gains are tuned empirically to ensure stable tracking performance under the tested operating conditions. While these parameters are not experimentally identified, they are reasonably assumed within typical ranges reported in the literature and are primarily used to validate the proposed energy-saving strategy. Their specific values can be flexibly adjusted in the simulation framework to examine the effectiveness of the control method under varying dynamic conditions, without affecting the qualitative conclusions of this study.

### 3.2. Energy Consumption Modeling of the VSJ Motors

Due to the strict portability and weight constraints of mobile robots, traditional AC power supplies cannot meet their flexible deployment requirements. DC servo motors are therefore widely used as the position motors in robotic joints. The motor selected here is EC 45 flat ∅ 42.8 mm, brushless, 70 W, with Hall sensors and cable. The power consumption of the main drive can be calculated using the following DC motor model:

(20) $$P_{e}=U_{z}I_{z}$$

where voltage $U_{z}$ and current $I_{z}$ are defined as:

(21) $$\left\{\begin{array}{l} I_{z}=\frac{t_{m}+\nu_{m}{\dot{\theta }}_{m}}{k_{t_{m}}}\\ U_{z}=R_{z}I_{z}+k_{{\dot{\theta }}_{m}}{\dot{\theta }}_{m}+L\frac{dI_{z}}{dt} \end{array}\right.$$

where $R_{z}$ and $L_{z}$ represent winding resistance and terminal inductance, respectively. $k_{t_{m}}$ and $k_{{\dot{\theta }}_{m}}$ represent the torque constant and speed constant of the position motor, respectively. The above parameters can be obtained from the motor manual provided by the manufacturer. The friction loss of motor bearings and brushes is expressed by the viscous damping coefficient $\nu_{m}$. Since the manufacturer usually does not specify the viscous damping coefficient of the motor, a simplified estimation is made based on the no-load operating characteristics of the motor, which can be expressed as:

(22) $$\nu_{m}=\frac{k_{t_{m}}I_{nl}}{\omega_{nl}}$$

where $I_{nl}$ and $\omega_{nl}$ are the no-load motor current and speed, respectively.

Then, the energy consumed during exercise is defined as:

(23) $$E_{pos}=\int P_{e}dt=\int U_{z}I_{z}dt$$

The stiffness adjustment transmission chain is relatively simple in configuration, consisting primarily of the adjustment motor, a reducer, the Archimedean spiral disk, and several rotating components. This transmission chain is decoupled from the main drive transmission, allowing independent regulation of joint rotational stiffness. The stiffness motor is primarily tasked with overcoming the resistive torque during stiffness reconfiguration rather than driving inertial loads. Based on this consideration, the stiffness adjustment motor is selected as the EC 22, ∅22 mm, brushless, 40 W. Its energy consumption is described by the same DC motor model as the main motor, as given in Equations (20)–(23). Consequently, the total energy consumption of the entire VSJ system is the sum of the energy consumed by the two motors:

(24) $$E=E_{pos}+E_{stiff}$$

where $E_{pos}$ and $E_{stiff}$ denote the energy consumption of the position drive motor and the stiffness motor, respectively.

### 3.3. Stiffness Control for VSJ

**Figure 8:**
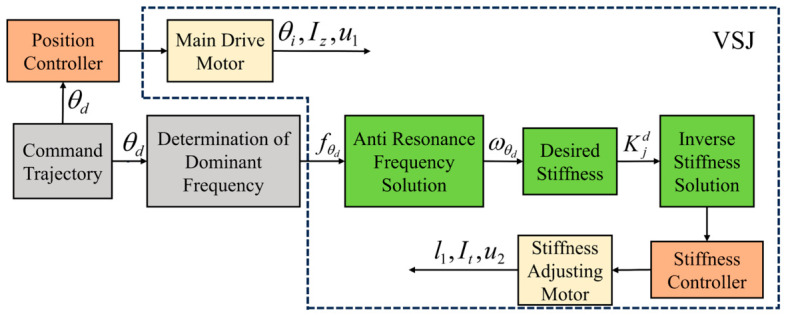
VSJ stiffness control strategy.

The stiffness control strategy is shown in Figure 8. Firstly, the command trajectory is used as the input to determine its main frequency as the expected anti-resonant frequency of the system. Secondly, the expected anti-resonant frequency is applied to the calculation of the expected joint rotational stiffness. Then, the optimal stiffness $K_{j}$ is obtained through the stiffness inverse solution model. Finally, the conversion from the target joint rotational stiffness $K_{j}$ to the effective length $l_{1}$ of the elastic cantilever is completed in combination with the characteristics of the elastic element. It provides the basis for the real-time control of the stiffness motor. In addition, due to the simplicity of the stiffness adjustment transmission chain, a PD controller is sufficient to cope with its underlying stiffness adjustment control.

#### 3.3.1. Anti-Resonance Frequency Solution

In order to achieve the goal of energy-saving control, it is necessary to keep the anti-resonance frequency of the system consistent with the dominant frequency of the task trajectory. According to the above dynamic model, the anti-resonant frequency of the system is determined by the inertia distribution at the output and the equivalent stiffness of the joint. The anti-resonant frequency varies with system stiffness, and this relationship can be derived from the characteristic equation. We adjust the joint rotational stiffness so that its inherent anti-resonance frequency is consistent with the task trajectory frequency, that is, $f_{a}=f_{\theta_{d}}$. Therefore,

(25) $$\omega_{a}=\sqrt{\frac{m_{p}gd_{p}+K_{j}}{J_{d}}}=\omega_{\theta_{d}}$$

The desired joint output stiffness is:

(26) $$K_{j}^{d}=J_{d}\omega_{\theta_{d}}^{2}-m_{p}gd_{p}=4\pi^{2}f_{\theta_{d}}^{2}J_{d}-m_{p}gd_{p}$$

#### 3.3.2. Inverse Stiffness Solution

In order to finally determine the stiffness $K_{j}^{d}$ by setting the stiffness motor, it must be converted into the effective spring length $l_{1}$ according to the characteristics of the elastic element. According to Equation (2), the effective spring length $l_{1}$ can be expressed as:

(27) $$l_{1}=\sqrt[{3}]{\frac{NEbh^{3}l_{2}^{2}\cos\varphi }{4K_{j}^{d}}}$$

where N is the number of leaf springs, taken as N=4.

The inverse solution model establishes the mapping between the target stiffness and the mechanical structure variables, providing the physical basis for stiffness control. The stiffness motor drives the adjusting disc, which in turn moves the sliding paddle radially, thereby changing the stress position and effective length of the elastic cantilever to achieve dynamic and continuous stiffness adjustment.

## 4. Simulation and Analysis for the Periodic Motion of VSJ

In periodic tasks, joints often perform repetitive motions. Unlike rigid actuators, VSJs adapt their stiffness to the motion state, preserving motion performance and enabling energy-saving control. Simulation analyses are performed using MATLAB and ADAMS. MATLAB optimizes the stiffness for the energy consumption model and control strategy, while ADAMS-MATLAB co-simulation validates energy consumption differences under different stiffness modes at the multi-body dynamics level.

### 4.1. Optimal Stiffness Adjustment Conditions

The motor model parameters are listed in Table 4. The power and energy required for the VSJ to simulate the common sinusoidal periodic trajectory motion are studied. The simplified dynamic model of the VSJ is established in MATLAB. The brushless DC servo motor is used as the driving source, and the input command trajectory is designed as:

(28) $$\theta_{d}=A\sin\left(2\pi f_{\theta_{d}}t\right)$$

**Figure 9:**
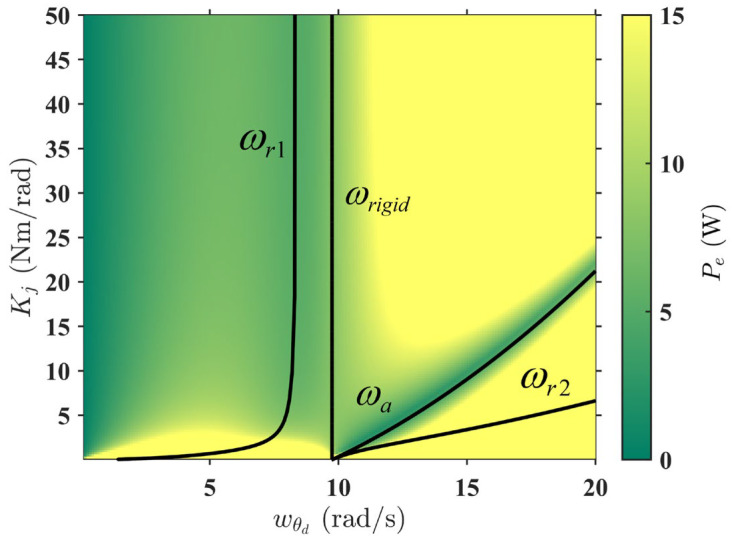
Mechanical peak power of the main drive link of VSJ under different trajectory frequencies and stiffness conditions.

Based on the DC motor model designed above, the peak power diagram of the VSJ position motor is simulated in MATLAB. Figure 9 shows the variation law of peak power of the VSJ main drive link under different trajectory frequency and stiffness conditions. The simulation results show that when the trajectory frequency approaches zero, the energy consumption of the position motor is maintained at a very low level regardless of the joint rotational stiffness level. In the actual working scene of the robot, the joints rarely complete the action of a large trajectory range at near-zero angular velocity. As the motion frequency increases, the energy demand of the main drive system rises significantly. This trend becomes more prominent under high-stiffness conditions, indicating that the coupling effect between trajectory frequency and joint rotational stiffness directly affects system energy consumption. Therefore, this coupling is a key factor to be considered in energy consumption optimization.

**Figure 10:**
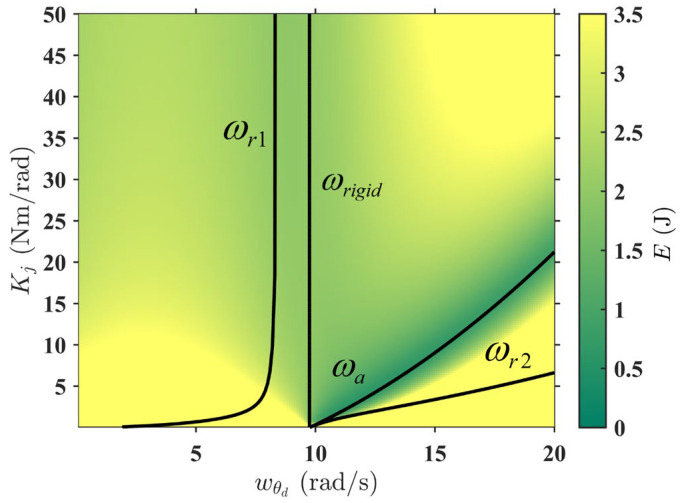
Single-cycle energy consumption of the main drive link of VSJ under different trajectory frequencies and stiffness conditions.

When the joint rotational stiffness is gradually increased and the angular velocity of the main drive matches the resonant frequency $\omega_{r1}$, defined in Equation (12), the corresponding frequency band exhibits a local minimum in power consumption. However, the power consumption at $\omega_{r2}$ decreases only slightly. The key factor causing this phenomenon is that when the output transmission system is in reverse operation, the transmission efficiency will be significantly reduced, which directly affects the optimization effect of power consumption. Furthermore, when the joint rotational stiffness is low and the motion frequency is high, the reference application value remains low. Under rigid transmission, when the motion angular frequency approaches a specific value $\omega_{ridid}$, the power consumption of the main drive link converges to a local minimum. The existence of an obvious interval shows that when the stiffness is adjusted to meet the specific condition $\omega_{\theta_{d}}=\omega_{a}$, the power required by the system can reach the global minimum in the corresponding periodic motion. Figure 10 also confirms this conclusion. In addition, when the stiffness approaches zero, increasing the motion frequency leads to a significant rise in system energy consumption. This indicates that a high-stiffness configuration is unsuitable for energy-saving scenarios in periodic motion.

### 4.2. Energy-Saving Motion Simulation Based on ADAMS and MATLAB

To verify the proposed energy-saving control strategy in a multi-body dynamics setting, an ADAMS-MATLAB co-simulation model is developed based on the optimal stiffness adjustment boundary derived from MATLAB. The VSJ periodic motion is simulated with full consideration of PID control, mechanism kinematics, and dynamic coupling.

#### 4.2.1. Co-Simulation Settings

**Figure 11:**
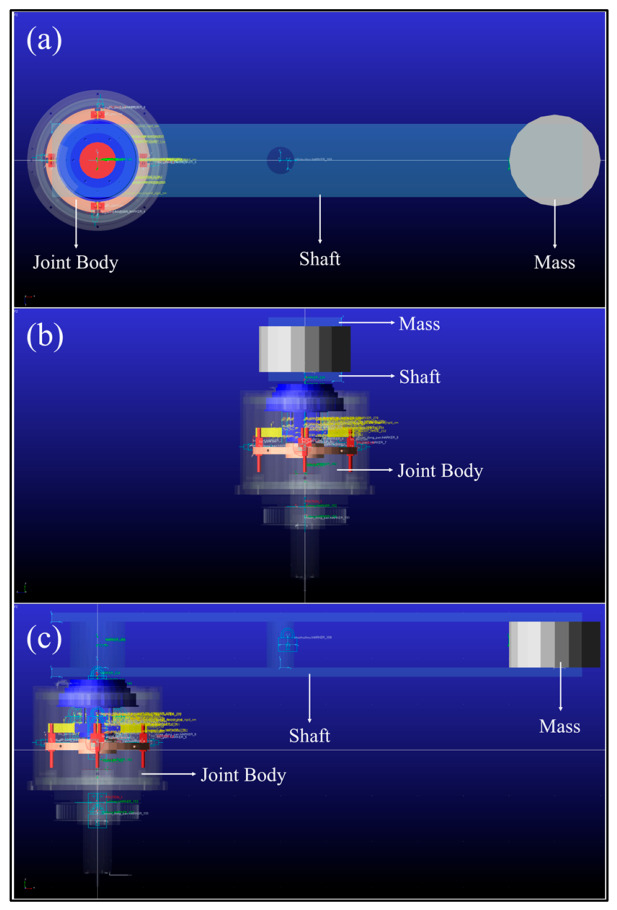
ADAMS three-dimensional model. (**a**) Top view. (**b**) Right view. (**c**) Front view.

The simplified model of the three-dimensional prototype is established in ADAMS, as shown in Figure 11a–c. The equivalent parameters of the main drive shaft frame, output end plate, leaf spring and other key components are configured. To highlight the energy-saving motion characteristics of the system, non-critical geometric details are appropriately simplified while maintaining consistency between the dynamic characteristics and the design model. The stiffness adjustment plate is removed, while the four mobile paddles and transmission plates used for stiffness adjustment are retained. Based on the above dynamic modeling results, the input inertia, output inertia, damping coefficient, and load parameters are configured. Additionally, to reduce model complexity and enhance the focus of energy consumption analysis, the global gravitational effect is not considered in the simulation.

**Figure 12:**
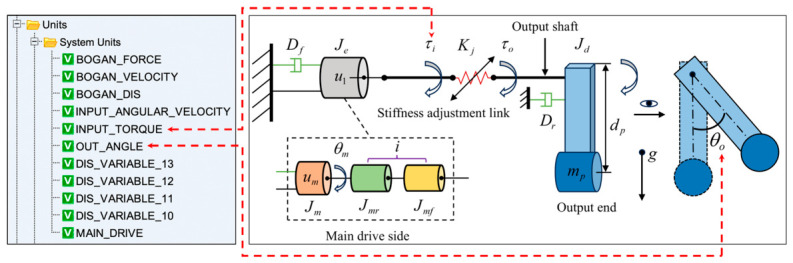
The setting and mapping relationship of ADAMS system unit for VSJ.

As shown in Figure 12, to implement the co-simulation between ADAMS and MATLAB, the required system units are first defined in ADAMS. Five of these units, MAIN_DRIVE and DIS_VARIABLE_10 to DIS_VARIABLE_13, are driven by MATLAB. They represent the spindle drive function and the radial displacement of the four sliding paddles on the drive plate during stiffness adjustment. Another six units, namely BOGAN_FORCE, BOGAN_VELOCITY, BOGAN_DIS, INPUT_ANGULAR_VELOCITY, INPUT_TORQUE, and OUT_ANGLE, represent the external force, velocity, displacement, input angular velocity, input torque, and output trajectory of the sliding paddle in the mechanical system, respectively. Based on these 11 system units, ADAMS and MATLAB perform real-time simulation data exchange.

**Figure 13:**
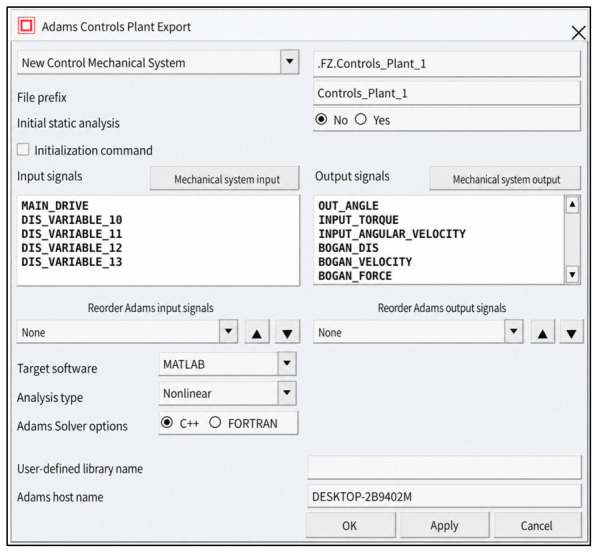
ADAMS export setting.

As illustrated in Figure 13, secondly, to establish the connection between MATLAB and ADAMS, the ADAMS Controls plug-in is selected. Within the plug-in interface, MATLAB is chosen as the target software, and C++ is selected as the solver for exporting the mechanical system model. This process generates a set of files that enable MATLAB to call and interact with the ADAMS mechanical model during co-simulation.

**Figure 14:**
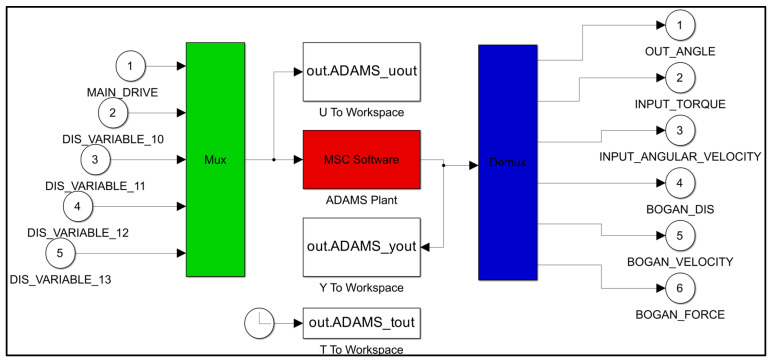
ADAMS control system unit in Matlab/Simulink.

Finally, as illustrated in Figure 14, the ADAMS mechanical system is exported, generating a corresponding function file that can be executed in MATLAB. Once this function file is run, the ADAMS mechanical system module is successfully loaded and made available for co-simulation within the MATLAB environment.

**Figure 15:**
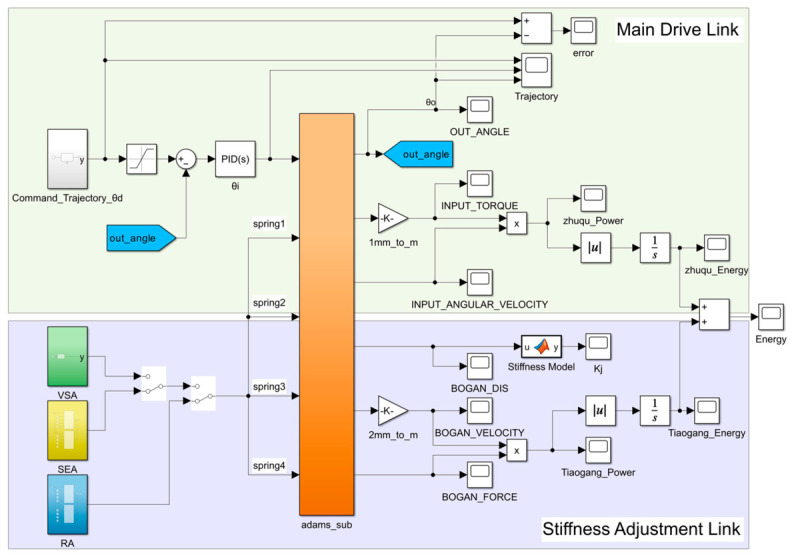
Co-simulation model in Matlab/Simulink.

As shown in Figure 15, in MATLAB/Simulink, the ADAMS mechanical system (orange module) is retained to establish the co-simulation model incorporating PID control. During simulation, the main drive PID controller generates a control signal and sends it to the ADAMS mechanical system. ADAMS, in turn, feeds the output angle back to the PID control module in real time, forming a closed-loop data interaction that ensures the output trajectory accurately tracks the task trajectory. Meanwhile, the stiffness control module computes the target stiffness based on the matching principle between the dominant frequency of the task trajectory and the anti-resonance frequency of the system. This target stiffness is then converted into the effective working length of the elastic cantilevers, serving as the input to the stiffness adjustment mechanism.

To ensure comparability of simulation results under different stiffness modes, the input trajectory, system parameters, and initial conditions in the co-simulation are kept consistent with the simplified model described above. Based on the simulation results above, the energy consumption comparison experiment scheme shown in Table 5 is designed. A periodic sinusoidal task trajectory is prescribed with a frequency range of 0.5–1.5 Hz, a duration of 10 s, and a constant amplitude of 2°. The main drive link starts from rest, while the rigid and constant stiffness modes complete their stiffness adjustment within 1 s. The total simulation time is set to 11 s, and the communication step between ADAMS and MATLAB is 0.0001 s. On this basis, three modes are established for comparative analysis:

(1) Rigid mode: characterizes the dynamics and energy consumption of a traditional rigid joint in periodic motion.
(2) Constant stiffness mode: investigates the system response and main drive energy consumption under a fixed stiffness condition.
(3) Variable stiffness mode: adjusts the joint rotational stiffness in real time according to the dominant frequency of the trajectory, matching the system’s anti-resonance frequency with the task frequency to validate the proposed energy-saving control method.

#### 4.2.2. Analysis of Simulation Results

**Figure 16:**
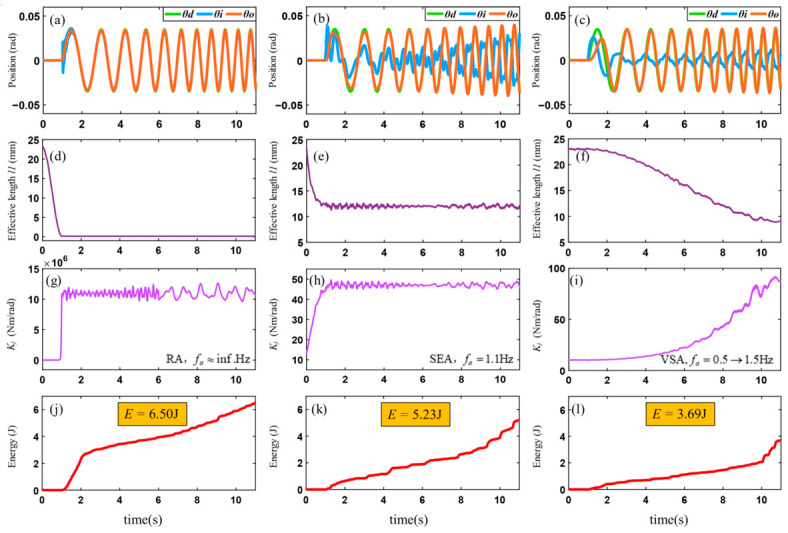
Simulation results of VSJ under RA, SEA, and VSA modes. (**a**–**c**) Trajectory tracking. (**d**–**f**) Variation in leaf spring effective length. (**g**–**i**) Stiffness. (**j**–**l**) Energy consumption.

As shown in Figure 16a,d,g,j, when the effective length of the leaf spring is adjusted to nearly 0 mm, the joint operates in rigid mode, with its theoretical stiffness approaching infinity. The actual stiffness, however, is determined by the specific structural parameters. Since the system starts from rest, a relatively large input oscillation occurs at the beginning. After this oscillation subsides, the trajectory tracking performance becomes satisfactory. At this stage, the natural frequency of the output is significantly higher than the frequency of the task trajectory. As the task trajectory frequency increases, the system energy consumption accumulates continuously, reaching approximately 6.50 J over the entire motion process.

As shown in Figure 16b,e,h,k, when the effective length of the leaf spring is controlled at 12 mm and the joint rotational stiffness is maintained at about $K_{j}=47.53\;\mathrm{Nm}/\mathrm{rad}$, the system is equivalent to the traditional constant stiffness mode (like a SEA), and the natural frequency of joint output is about 1.1 Hz. As the frequency of the task trajectory signal changes, the joint input $\theta_{i}$ first decreases from the initial larger value to a lower level, and then increases to a higher level. The lowest peak appears at about t=6 s. At this time, the joint operates in a state where the natural frequency of the output is equal to the task trajectory frequency $f_{\theta_{d}}=f_{a}$. The results show that the stiffness fluctuation is relatively small here, because the phase difference between the input and output trajectories is about 90°, and the coupling effect between the stiffness regulating mechanism and the main drive is slight. In other time periods, the natural frequency of the system deviates significantly from the task trajectory frequency, resulting in a relatively large corresponding input. The energy consumed by VSJ in the whole process under SEA mode is about 5.23 J.

As shown in Figure 16c,f,i,l, when the joint is in VSA mode, the system stiffness is continuously adjusted over time to match the task trajectory frequency in real time. The simulation results show that only a small input $\theta_{i}$ is enough to meet the trajectory tracking of the output. In this mode, the whole process of the task meets $f_{\theta_{d}}=f_{a}$, the fluctuation of joint rotational stiffness is slight. The required input $\theta_{i}$ is also kept at a low level, which only increases slightly with the increase in task trajectory frequency. The energy consumed by VSJ in the whole process under VSA mode is only about 3.69 J.

**Figure 17:**
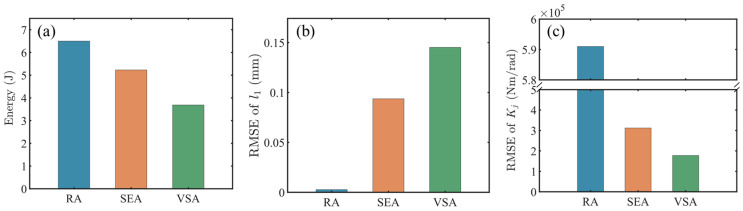
RMSE comparison of VSJ under RA, SEA, and VSA modes. (**a**) Energy consumption. (**b**) The effective spring length. (**c**) Stiffness.

As shown in Figure 17, the three working modes exhibit significant differences in total energy consumption, as well as in the RMSE of the leaf spring effective length and the joint rotational stiffness. As shown in Figure 17a, the RA mode exhibits the highest total energy consumption among the three working modes, followed by the SEA mode and then the VSA mode. Figure 17b reveals distinct characteristics in the RMSE of the leaf spring effective length across the three modes. In RA mode, the effective length remains essentially near zero, resulting in an RMSE of nearly zero. In SEA mode, the effective length is held constant, yielding a moderate RMSE of approximately 0.09 mm. In contrast, the VSA mode continuously adjusts the effective length of the leaf spring during motion to adapt to variations in trajectory frequency, leading to a relatively large RMSE of about 0.14 mm. This reflects the active regulatory role of the stiffness adjustment mechanism in stiffness control. Figure 17c presents the RMSE of joint equivalent stiffness. In RA mode, the joint operates in an approximately rigid state, and its stiffness RMSE is substantially higher than that of the other two modes. The SEA mode maintains a fixed stiffness, with an RMSE of approximately 3.1 Nm/rad. The VSA mode achieves the smallest stiffness RMSE, approximately 1.8 Nm/rad, demonstrating its superior stiffness regulation capability. It also proves that the synchronous stiffness adjustment strategy mentioned above achieves good compliance between joint motion and command frequency in the energy-saving motion control process.

**Figure 18:**
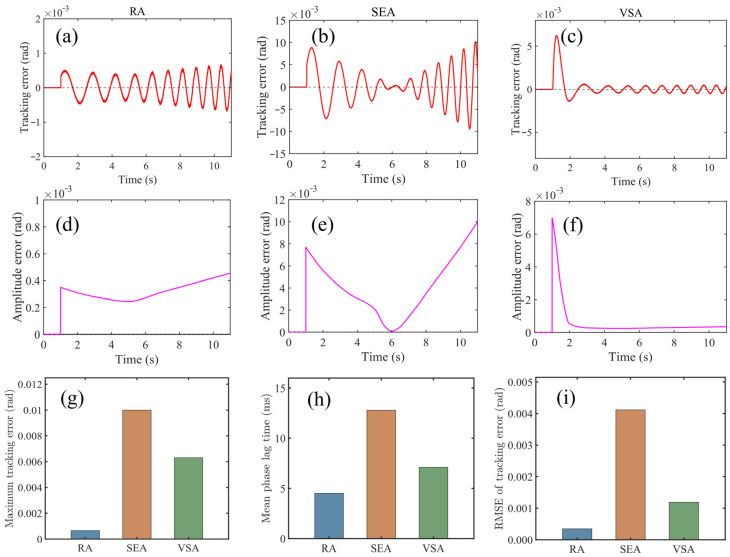
Trajectory tracking performance comparison of VSJ under RA, SEA, and VSA modes. (**a**–**c**) Tracking errors. (**d**–**f**) Amplitude error curves. (**g**) Maximum tracking error. (**h**) Mean phase lag time. (**i**) RMSE of tracking error.

As shown in Figure 18, the three working modes exhibit distinct trajectory tracking characteristics. Figure 18a–c show the tracking errors under RA, SEA, and VSA modes, respectively. The RA mode maintains a small tracking error throughout the motion, with only slight oscillations as the frequency increases. In contrast, the SEA mode presents a relatively large tracking error: the error decreases to around 6 s due to the resonance effect, but increases again after approximately 7 s as amplitude overshoot becomes more pronounced. The VSA mode shows a noticeable initial tracking error during 1–1.5 s caused by transient stiffness adjustment and phase lag, while the tracking performance improves rapidly after 2 s.

Figure 18d–f further illustrate the amplitude error characteristics, which are fitted by the envelope of the maximum amplitude of tracking errors. As shown in Figure 18d, in RA mode, the tracking error amplitude decreases slightly during the initial stage (t < 5 s) due to system start-up transients, and then gradually increases with the rising oscillation frequency, yet the overall fluctuation remains small, indicating high position tracking accuracy of the VSJ under rigid transmission. As shown in Figure 18e, in SEA mode, the introduction of the elastic element leads to a notable increase in tracking error. Since the joint output-side natural frequency matches the task frequency near 6 s under the current stiffness setting, the error amplitude exhibits a V-shaped trend, which validates the effectiveness of the co-simulation. As shown in Figure 18f, in VSA mode, although the tracking error is relatively large initially due to disturbances from stiffness adjustment, it decreases rapidly and stabilizes after 2 s once the energy-saving condition is satisfied.

As summarized in Figure 18g–i, the maximum tracking errors of the RA, SEA, and VSA modes are approximately 0.00065 rad, 0.0100 rad, and 0.0063 rad, respectively. The mean phase lag times of the RA, SEA, and VSA modes are approximately 4.5 ms, 12.8 ms, and 7.1 ms, respectively. The RMSE values of tracking error are approximately 0.000345 rad, 0.00412 rad, and 0.00119 rad, respectively. Although the RA mode shows the smallest tracking error and phase lag due to its rigid transmission characteristics, it produces higher energy consumption. In contrast, the VSA mode provides a better balance between tracking error reduction, motion synchronization, and energy efficiency.

## 5. Discussion

The results demonstrate that the proposed method can reduce energy consumption in periodic motions when the joint anti-resonance frequency is aligned with the task trajectory frequency. However, several practical factors and inherent limitations of this approach warrant further discussion to provide a balanced assessment of its applicability.

### 5.1. Practical Mechanical Factors Affecting Energy-Saving Performance

In practical applications, friction in the spiral groove and between sliding contact surfaces, which is assumed ideal in the dynamic model, consumes part of the actuator energy and may cause dead zones during forward and reverse rotations. During stiffness adjustment, clearance-induced vibrations may require additional motor energy to suppress, thereby increasing the actual energy consumption beyond the simulated values. Leaf springs exhibit significant hysteresis due to interleaf friction, leading to additional energy dissipation that reduces the conservation of stored elastic energy. Cyclic loading may also induce fatigue and gradual stiffness degradation over extended operation, potentially altering the tuned anti-resonance frequency and compromising the long-term energy-saving effectiveness.

Mechanical clearances and backlash in the joint transmission chain introduce nonlinear dynamic behaviors that can affect stiffness adjustment precision and tracking accuracy. The stiffness adjustment mechanism has an inherent response time, typically on the order of 50 to 200 milliseconds for stiffness changes, plus additional time for position stabilization, which may cause phase lag between the desired and actual stiffness trajectories in tasks with rapidly varying frequencies.

### 5.2. Applicability Bandwidth and Operational Boundaries

Beyond the mechanical factors discussed above, the stiffness-frequency matching strategy itself has inherent operational limitations that merit careful consideration. The energy-saving effect is most pronounced when the task trajectory is a periodic signal with a well-defined dominant frequency, such as a sinusoidal motion. As shown in Figure 9, the energy consumption exhibits a distinct minimum at the frequency where the anti-resonance frequency matches the trajectory frequency $\omega_{a}$, but this minimum is relatively narrow. Deviations from the optimal condition result in rapidly increasing energy consumption, indicating limited benefits for motions with significant frequency variations or broad-spectrum characteristics.

The optimal stiffness derived from the frequency matching condition Equation (24) depends on both the trajectory frequency and the load inertia. Variations in payload would shift the optimal stiffness and degrade performance unless adaptive re-tuning is implemented. For non-sinusoidal periodic signals containing higher harmonic components, the proposed strategy becomes less effective because the actuator must provide torque at multiple frequencies simultaneously, and the stiffness cannot satisfy the anti-resonance condition for all harmonics. Furthermore, the stiffness reduction that minimizes energy consumption at the anti-resonance condition also lowers the joint mechanical impedance, which may compromise disturbance rejection and tracking accuracy under external perturbations. This represents a trade-off between energy efficiency and control performance that must be balanced in application-specific scenarios.

### 5.3. Applicability and Outlook

The proposed method is most suitable for tasks with stable, sinusoidal periodic trajectories and constant payloads, whereas its benefits may be compromised in scenarios involving frequency variations, amplitude changes, non-sinusoidal waveforms, or significant mechanical imperfections. Future research may also explore adaptive stiffness tuning strategies that can track varying optimal conditions in real time, as well as the integration of disturbance observers or robust control techniques to mitigate the trade-off between energy efficiency and tracking performance under uncertain operating conditions.

## 6. Conclusions

This paper presents the design, dynamic modeling, and energy-saving control of a leaf spring-based variable stiffness joint (VSJ) with an Archimedean spiral groove mechanism. Inspired by the human elbow joint’s resonance strategy during periodic arm swing, it shows that matching joint rotational stiffness to align the system’s anti-resonance frequency with the task trajectory’s dominant frequency significantly reduces periodic motion energy consumption. MATLAB simulations reveal an optimal stiffness condition in variable stiffness mode that lowers peak power and energy demand, unlike rigid mode.

Co-simulation results confirm that the VSJ in variable stiffness mode consumes only 3.69 J, versus 6.50 J (rigid) and 5.23 J (fixed-stiffness SEA), saving 43.2% and 29.5%, respectively. Lower RMSE values of stiffness and effective length indicate better dynamic matching between joint natural frequency and task frequency. These findings validate that the bioinspired stiffness-frequency matching principle observed in human elbow function translates effectively to a robotic VSJ for energy-efficient periodic motion.

It is worth noting that the current conclusions are drawn from a comprehensive co-simulation framework that incorporates realistic joint dynamics and control nonlinearities, providing a reliable theoretical basis for the proposed energy-saving strategy. While experimental validation with a physical prototype is not within the scope of this paper, the simulation results, particularly the consistent energy reduction across multiple operating modes and the improved dynamic matching metrics, offer strong evidence for the effectiveness of the stiffness-frequency matching principle. The quantitative comparisons and performance trends presented herein are expected to be reproducible in practice, and the modeling methodology lays a solid groundwork for future experimental investigations. Future work includes prototype fabrication, experimental validation, model refinement (friction, clearance, nonlinear deformation, actuator delay), and application of the optimization strategy to more complex tasks and variable loads.

## Figures and Tables

**Table 1:** Comparison between representative spring-based VSJ/VSA systems and the proposed design.

Stiffness Adjustment Principle	Stiff. Range (Nm/rad)	Structural Complexity	Validation Method	Energy-Saving Strategy	Ref.
Variable lever force point using Archimedean spiral and double-layer slide mechanism	17.2–1065	High (Motors coupled)	Simulation and experiments	Low-power stiffness regulation	[8]
Leaf-spring length adjustment with cam-roller and link mechanism	4–435	High+	Simulation and experiments	Passive energy storage and release	[9]
Adjustable torsion spring equilibrium position	Narrow	Medium	Single simulation	Passive energy storage and release	[10]
Leaf-spring length adjustment with ball-screw	0–1787	Medium	Hopping experiments	Low-power stiffness regulation and passive energy reuse	[11]
Cam-leaf spring mechanism with adjustable spring length	50–964	Medium	Simulation and experiments	Low-power stiffness regulation	[12]
Parallel variable stiffness spring with adjustable pivot	1.9–29.3	Medium	Oscillation tests	Resonance tuning for oscillation	[13]
Electromagnetic force attraction and friction control	Narrow	Low	Lack of targeted testing	PWM current control	[28]
Archimedean spiral grooves adjust elastic-cantilever effective length	11–∞	Medium-	Co-simulation	Anti-resonance frequency matching	VSJ

**Table 2:** VSJ design parameters.

Parameter	Value	Unit
Diameter	140	mm
Height	114	mm
Weight	4	kg
Stiffness adjustment range	11~∞	Nm/rad
Leaf spring width *b*	10	mm
Leaf spring thickness *h*	0.3	mm
Young’s modulus of leaf spring *E*	200	GPa

**Table 3:** Key parameters of VSJ dynamic system.

Parameter	Value	Unit
Lower limit of elastic cantilever length $l_{\min}$	0	mm
Upper limit of elastic cantilever length $l_{\max}$	23	mm
Input inertia $J_{e}$	4.166 × 10^−3^	kg m^2^
Output inertia $J_{d}$	0.983	kg m^2^
Gear ratio of reducer i	100	-
Damping coefficient $D_{r}$	1 × 10^−2^	Nm s/rad
Damping coefficient $D_{f}$	4.1 × 10^−2^	Nm s/rad
Reduction mechanism efficiency $\eta_{r}$	0.75	-
Output inertia $J_{d}$	0.983	kg m^2^
Load weight $m_{p}$	3	kg
Load arm $d_{p}$	0.5	m
Gravitational acceleration g	9.81	m/s^2^
Position gain $k_{1}$	1.1 × 10^−4^	Nm/rad
Speed gain $k_{2}$	3.5 × 10^−3^	Nm/s/rad
Acceleration gain $k_{3}$	550	Nm/s^2^/rad
Jerk gain $k_{4}$	50	Nm/s^3^/rad

**Table 4:** VSJ motor parameters.

Parameter	Position Motor	Stiffness Motor	Unit
Torque constant $k_{t_{m}}$	40.4	6.49	mNm/A
Speed constant $k_{{\dot{\theta }}_{m}}$	236	1470	rpm/V
Resistance constant $R_{z}$	0.573	0.641	Ω
Terminal inductance $L_{z}$	0.301	0.055	mH
No load current $I_{nl}$	270	144	mA
No load speed $\omega_{nl}$	5600	35,200	rpm
Maximum efficiency η	84.7	88	%

**Table 5:** Energy consumption comparison experimental design for VSJ.

Parameter	Unit	Compare Settings		
Command trajectory $\theta_{d}$	°	$2\sin\left(2\pi f_{\theta_{d}}t\right)$		
Working mode	-	RA	SEA	VSA
Trajectory frequency $f_{\theta_{d}}$	Hz	-	-	0.5 + 0.1(t − 1)
Output natural frequency $f_{a}$	Hz	≈inf.	1.1	0.5 + 0.1(t − 1)
Joint rotational stiffness $K_{j}$	Nm/rad	≈inf.	47.53	96–11.09
Effective spring length $l_{1}$	mm	0	12	9–23

## Data Availability

The data that support the findings of this study can be obtained from the corresponding author upon request.
